# Fenton-Like Oxidation of Antibiotic Ornidazole Using Biochar-Supported Nanoscale Zero-Valent Iron as Heterogeneous Hydrogen Peroxide Activator

**DOI:** 10.3390/ijerph17041324

**Published:** 2020-02-19

**Authors:** Yanchang Zhang, Lin Zhao, Yongkui Yang, Peizhe Sun

**Affiliations:** 1 School of Chemical Engineering and Technology, Tianjin University, Tianjin 300350, China; zhangyanchang@tju.edu.cn; 2 School of Environmental Science and Engineering, Tianjin University, Tianjin 300350, China; ykyang@tju.edu.cn (Y.Y.); sunpeizhe@tju.edu.cn (P.S.)

**Keywords:** nanoparticles, biochar, wastewater treatment, ornidazole

## Abstract

Biochar (BC)-supported nanoscale zero-valent iron (nZVI-BC) was investigated as a heterogeneous Fenton-like activator to degrade the antibiotic ornidazole (ONZ). The characterization of nZVI-BC indicated that BC could enhance the adsorption of ONZ and reduce the aggregation of nZVI. Thus, nZVI-BC had a higher removal efficiency (80.1%) than nZVI and BC. The effects of parameters such as the nZVI/BC mass ratio, pH, H_2_O_2_ concentration, nZVI-BC dose, and temperature were systematically investigated, and the removal of ONZ followed a pseudo-second-order kinetic model. Finally, possible pathways of ONZ in the oxidation process were proposed. The removal mechanism included the adsorption of ONZ onto the surface of nZVI-BC, the generation of •OH by the reaction of nZVI with H_2_O_2_, and the oxidation of ONZ. Recycling experiments indicated that the nZVI-BC/H_2_O_2_ system is a promising alternative for the treatment of wastewater containing ONZ.

## 1. Introduction

Ornidazole (ONZ), which is a third-generation 5-nitroimidazole antibiotic, is widely used to treat infections owing to its excellent activity against anaerobic bacteria [1,2]. Compared with other antibiotics, ONZ has a longer elimination half-life and greater capacity to penetrate into lipidic tissues, which makes it a good choice in dental and gastrointestinal surgery [3]. However, it poses a risk to humans and wildlife if it is discharged into the environment owing to its potential genotoxic, carcinogenic, and mutagenic properties [4,5]. The presence of ONZ in the environment, such as surface water and ground water, was investigated [6]. Wastewater containing ONZ must be treated before entering the environment. However, the high aqueous solubility (4.33 g/L) and low biodegradability of ONZ make it challenging to remove ONZ via traditional treatment techniques. A few studies were performed to eliminate ONZ using photocatalysts [7,8]. However, the complex procedures of these methods may limit their large-scale application, and it is desirable to develop more low-cost and efficient technologies to remove ONZ from wastewater.

Advanced oxidation processes (AOPs) are widely employed for the treatment of organic contaminants. Among the AOPs, the Fenton reaction is one of the most popular and effective methods for dealing with many pollutants [9,10]. The conventional Fenton reaction involves ferrous salts reacting with hydrogen peroxide under acidic conditions, generating hydroxyl radicals (•OH, oxidation potential of 2.8 eV) which can oxidize a wide range of contaminants rapidly. However, Fe^3+^ is easily precipitated as Fe(OH)_3_ sludge, which causes secondary pollution and reduces the degradation efficiency [11]. Nanoscale zero-valent iron (nZVI) was proposed as an alternative iron source to activate H_2_O_2_. nZVI can supply Fe^2+^ continuously and produce less sludge owing to ferric ion recycling in the system [12].

(1) $${\mathrm{Fe}}^{2+}+H_{2}O_{2}\to {\mathrm{Fe}}^{3+}+•\mathrm{OH}+{\mathrm{OH}}^{-}.$$

(2) $$2{\mathrm{Fe}}^{3+}+\mathrm{Fe}\to 3{\mathrm{Fe}}^{2+}.$$

The heterogeneous Fenton process using nZVI was employed for the degradation of organic contaminants, particularly in the treatment of personal care products [13,14]. However, nZVI was easily oxidized in the presence of oxygen and tended to aggregate because of high surface energy [15], which had detrimental effects on the stability and catalytic activity. To overcome these drawbacks, porous materials such as zeolites [16], mesoporous carbon [17], montmorillonite [18], and mesoporous silica [19] were used as the supporting materials for nZVI immobilization, which reduced the aggregation and enhanced its transport. 

Biochar (BC) is a carbon-rich material with a large surface area and a porous structure, and it is produced by the thermal conversion of biomass under oxygen-limited conditions. Recently, the application of BC in water treatment attracted considerable attention because BC is low-cost and abundant, and it has extraordinary adsorption properties for removing organic contaminants [20]. Several studies reported that BC with a large surface area can serve as an effective supporter of nZVI [21,22]. A biochar-supported nanoscale zero-valent iron composite was successfully used as a persulfate activator for removing nonylphenol and trichloroethylene [22,23]. In the present study, we attempted to degrade ONZ and analyze the related mechanism using an nZVI-BC/H_2_O_2_ system owing to its strong oxidative capacity. So far, there are no reports on this. Therefore, the application of the nZVI-BC/H_2_O_2_ system for the removal of ONZ is of great importance.

The objectives of this study were as follows: (1) to synthesize and characterize the nZVI-BC composites, (2) to investigate the effects of the initial pH, H_2_O_2_ concentration, nZVI-BC dose, and temperature on the degradation of ONZ, (3) to assess the reusability and stability of nZVI-BC, and (4) to clarify the removal mechanism and possible pathways of ONZ in the nZVI-BC/H_2_O_2_ system.

## 2. Experimental Methods

### 2.1. Chemicals

ONZ (>98%), sodium borohydride (NaBH_4_, >98%), ferrous sulfate heptahydrate (FeSO_4_·7H_2_O, >99%), hydrogen peroxide (H_2_O_2_, 30% aqueous solution), *tert*-butyl alcohol (TBA, >99.5%), and absolute ethyl alcohol were purchased from Aladdin, Shanghai. All the chemicals were analytical-grade, and ultra-pure water (18.2 MΩ) was used in this study.

### 2.2. Preparation of nZVI-BC

BC was produced via pyrolysis of bamboo sawdust. The bamboo sawdust was collected from a farm in Gongyi (Henan Province, China), washed several times, and dried at 80 °C overnight. The raw material was crushed and passed through a 40-mesh sieve. Then, it was placed into tightly filled ceramic crucibles and pyrolyzed in a muffle furnace at 600 °C under an oxygen-limited environment for 2 h. The obtained BC was treated with 1 M HCl for 2 h to remove inorganic components and washed with distilled water several times to remove any residual acids.

The nZVI-BC was synthesized via a conventional liquid-phase reduction method. In brief, 4.96 g of FeSO_4_·7H_2_O and BC (0.5, 1.0, 2.0, 3.0 g) were added to 100 mL of a water–ethanol solution (*w*/*w* = 3:2) with stirring for 60 min. Then, 100 mL of a 0.36 M NaBH_4_ solution was added to this mixture dropwise under vigorous mechanical agitation. Subsequently, the mixture was stirred for 1 h. The black precipitant was collected via vacuum filtration and quickly washed with absolute ethyl alcohol and water three times each. Then, it was dried under vacuum conditions overnight and stored in an N_2_ atmosphere for further use. The four types of nZVI-BC composites with Fe^0^/BC mass ratios of 2:1, 1:1, 1:2, and 1:3 were denoted as nZVI-BC_1_, nZVI-BC_2_, nZVI-BC_3_, and nZVI-BC_4_, respectively. nZVI was prepared via the same method without the addition of BC. The process can be described by the following reaction [13,24]:

(3) $${\mathrm{Fe}}^{2+}+2{\mathrm{BH}}_{4}^{-}+6H_{2}O\to \mathrm{Fe}+2B{\left(\mathrm{OH}\right)}_{3}+7H_{2}↑.$$

### 2.3. Characterization

The morphological characteristics were analyzed using scanning electron microscopy (SEM, S-4800, Hitachi Company, Japan) and transmission electron microscopy (TEM, JEM-1200EX, JEOL Ltd., Japan). The surface structure and composition were examined via X-ray diffraction (XRD) analysis using a Bruker D8 Advanced diffractometer with Cu/Kα radiation (λ = 1.5406 Å), as well as X-ray photoelectron spectroscopy (XPS, ESCALAB 250Xi, Thermo Corporation, USA). The specific surface area and the pore structure were determined using a Brunauer–Emmett–Teller (BET) analyzer (ASAP 2460, Micromeritics, USA). The Fourier-transform infrared (FTIR) spectra were acquired using a Nicolet 380 spectrometer. The zeta potentials were measured using a Zetasizer Nano ZS 90 (Malvern, UK).

### 2.4. Batch Experiments

All batch experiments were performed in 500-mL conical flasks sealed with rubber plugs in a water bath at a speed of 400 rpm. To initiate the experiments, different amounts of H_2_O_2_ and nZVI-BC were added to 300 mL of a 100 mg/L ONZ solution. The effects of the initial solution pH (2.0–6.0), initial H_2_O_2_ concentration (4–24 mM), nZVI-BC dose (0.05–0.40 g/L), and temperature (15–45 °C) were investigated by changing one factor while keeping the others constant. The pH of the ONZ solution was adjusted using 0.1 M H_2_SO_4_ or NaOH. At regular intervals, an aliquot amount (3 mL) of the reaction solution was withdrawn, filtered through a 0.22-μm membrane film, and quenched with TBA immediately for further analysis. All batch experiments were performed in triplicate to ensure reproducibility, and the relative errors were controlled within ±5%. The removal efficiency of ONZ was calculated as follows:

(4) $$R\left(\%\right)=\frac{C_{0}-C_{t}}{C_{0}}\times 100,$$

where C_0_ and C_t_ (mg/L) represent the ONZ concentrations at the initial time and time t, and R (%) represents the ONZ degradation efficiency.

### 2.5. Analytical Methods

The concentration of ONZ was measured in a Waters e2695 HPLC system equipped with an Agilent C18 column (4.6 × 250 mm, 5 μm). The mobile phase was composed of methanol and water (20:80, *v*/*v*) with a flow rate of 1.0 mL/min. The ONZ was measured at a wavelength of 318 nm, and the column temperature was 30 °C. An ultraviolet–visible light (UV–Vis) spectrophotometer (D6000, Hach, USA) was used to obtain the UV spectra of the ONZ solution. The chemical oxygen demand (COD) and total organic carbon (TOC) were measured using a COD tester (DRB200, Hach, USA) and a TOC analyzer (TOC-VCPH, Shimadzu Corporation, Japan), respectively. The pH of the solution was measured using a Mettler-Toledo pH meter. An ion chromatograph (DX600, Dionex, USA) was used to detect the inorganic ions. The carbon content was determined by element analysis (Vario EL Ⅲ, Elementar, Germany). The iron content was determined with inductively coupled plasma atomic emission spectrometry (ICP-OES, ICPOES730, Agilent, USA) after HNO_3_ digestion.

## 3. Results and Discussion

### 3.1. Characterization of nZVI-BC

The BET specific surface area and pore volume of the BC and the different nZVI-BC composites are presented in Table 1. The specific surface area and pore volume of the nZVI-BC were significantly increased compared with those of the nZVI. The fraction of nZVI loaded on BC decreased from 50.8% to 21.6% when the Fe^0^/BC mass ratio increased from 2:1 to 1:3 (Table S1, Supplementary Materials). The specific surface area of the nZVI-BC increased with an increase in the BC proportion. This may be because the BC provided enough sites for iron particles, and higher BC content favored the dispersion of nZVI. However, a further increase of BC content resulted in a lower BET surface area value due to the increasing aggregation of BC sheets [25,26].

**Figure 1:**
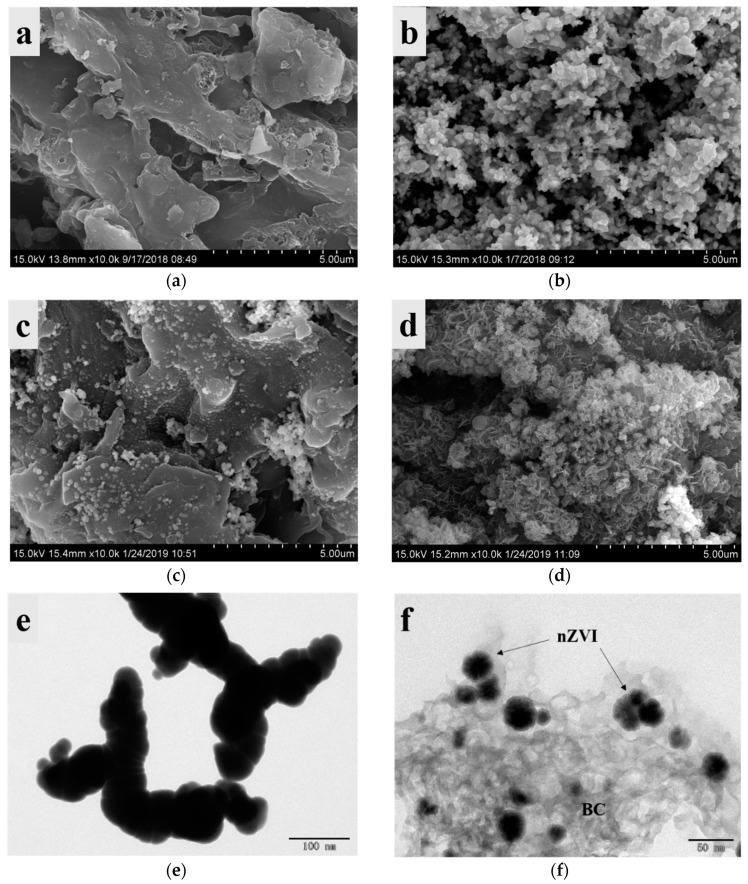
SEM images of biochar (BC) (**a**), nanoscale zero-valent iron (nZVI) (**b**), and nZVI-BC_3_ before (**c**) and after (**d**) reaction. TEM images of nZVI (**e**) and nZVI-BC_3_ (**f**).

The SEM images revealed that the BC had a microporous structure (Figure 1a), which was conducive to the impregnation of nZVI. The nZVI particles aggregated as clusters owing to the van der Waals forces (Figure 1b) [27]. In contrast, numerous small globular particles were distributed well on the BC surface (Figure 1c), indicating that iron nanoparticles successfully attached onto the BC without significant aggregation. The particles lost their spherical shape after the reaction (Figure 1d), suggesting the corrosion of Fe^0^ and the formation of iron-oxide products on the BC. Additionally, a TEM image indicated that the particles were attached uniformly onto the surface of BC, and that the size of nZVI-BC_3_ was approximately 20–50 nm (Figure 1f), which was in the nanoscale range. However, the nZVI particles were densely distributed, and their size was larger than that of nZVI-BC_3_ (Figure 1e). The results indicated that the BC reduced the aggregation of nanoparticles and effectively supported the nZVI. 

**Figure 2:**
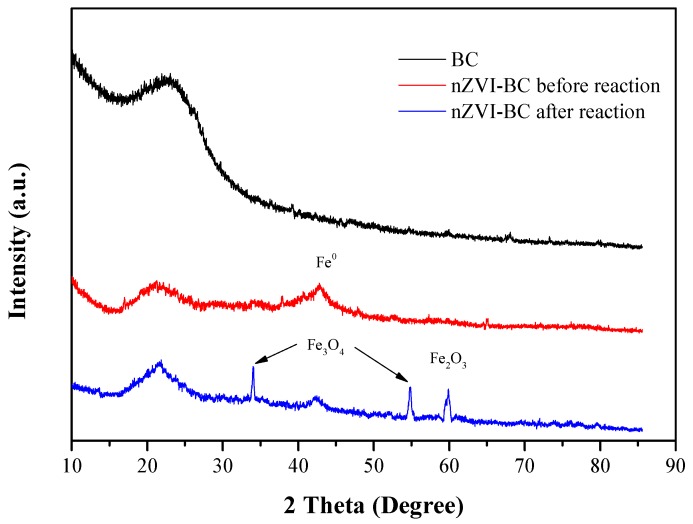
X-ray diffraction (XRD) patterns of BC, and nZVI-BC_3_ before and after reaction.

The XRD analysis results for the BC and nZVI-BC_3_ before and after the reaction are presented in Figure 2. The broad peak at 2θ = 22.4° indicates the presence of amorphous BC [28,29], corresponding to a *d*-spacing of 0.4 nm according to Bragg’s Law. The large *d*-spacing was attributed to the presence of C–O and O=C–O [30]. For nZVI-BC_3_, the peak at 2θ = 44.9°confirmed the presence of Fe^0^ [31]. Furthermore, a peak at 22.4° was observed, but the intensity was weakened, indicating that nZVI was successfully loaded in the pores of the BC. For the reacted nZVI-BC_3_, characteristic peaks of Fe_3_O_4_ (2θ = 35.4°/57.2°) and Fe_2_O_3_ (2θ = 62.7°) appeared [13]. The peak of Fe0 was still present, but was less pronounced than that for the fresh nZVI, indicating that some of the Fe^0^ was consumed in the reaction.

**Figure 3:**
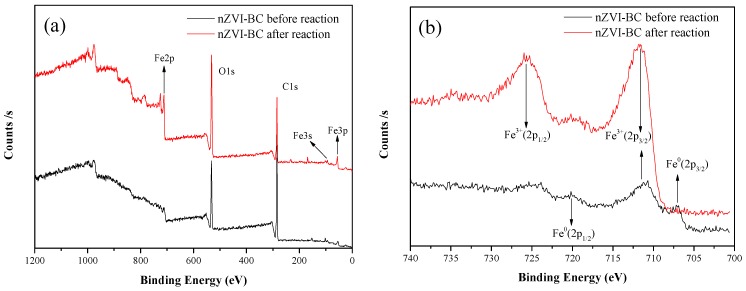
X-ray photoelectron spectroscopy (XPS) patterns from the full survey (**a**), and scan of Fe 2*p* (**b**) for nZVI-BC_3_ before and after reaction.

Figure 3 presents the XPS patterns of nZVI-BC. For fresh nZVI-BC, the peaks at 706.9 and 720.2 eV indicated the presence of Fe^0^ in nZVI-BC, and the peak at 711.1 eV indicated Fe^3+^ (Fe_2_O_3_) [32]. These results suggest that the iron particles were covered by an oxide film, which formed a core–shell structure [33]. For the reacted nZVI-BC, the peaks at 711.1 and 725.1 eV corresponded to Fe_2_O_3_. Moreover, the O 1*s* feature peaks at 530 eV in this region confirmed that Fe_2_O_3_, Fe_3_O_4_, and FeOOH all existed on the surface of nZVI before and after the reaction [34], and the iron hydroxides were dehydrated to oxides. These results indicate that the nZVI-BC was oxidized after the reaction, in accordance with Equations (5)–(8), which is consistent with the XRD results [13,35]. BC was mainly represented by C 1*s* with a peak of 284.8 eV.

(5) $$4{\mathrm{Fe}}^{2+}+4H^{+}+O_{2}\to 4{\mathrm{Fe}}^{3+}+2H_{2}O.$$

(6) $${\mathrm{Fe}}^{3+}+2H_{2}O\to \mathrm{FeOOH}+3H^{+}.$$

(7) $$2\mathrm{FeOOH}\to {\mathrm{Fe}}_{2}O_{3}+H_{2}O.$$

(8) $$6{\mathrm{Fe}}^{2+}+O_{2}+6H_{2}O\to 2{\mathrm{Fe}}_{3}O_{4}+12H^{+}.$$

**Figure 4:**
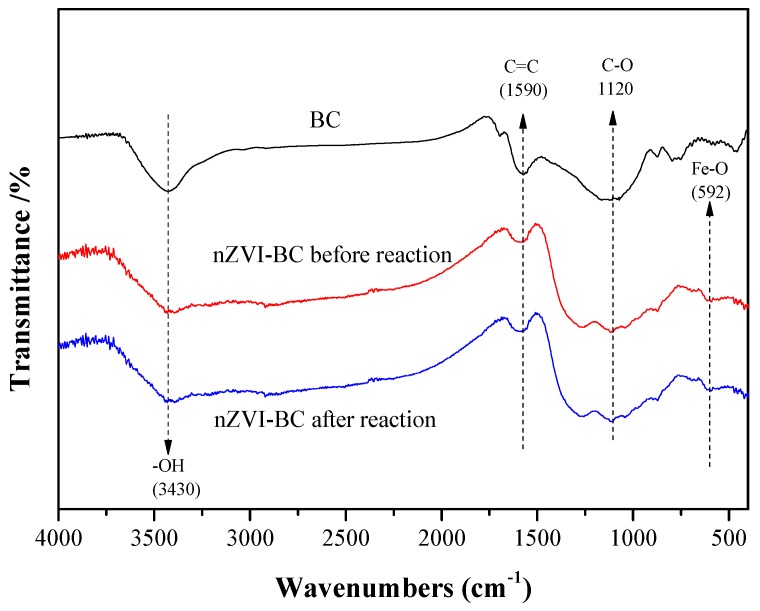
Fourier-transform infrared (FTIR) spectra of BC, and nZVI-BC_3_ before and after reaction.

The FTIR spectra of BC and nZVI-BC are presented in Figure 4. BC is a carbonaceous material with abundant functional groups [23]; therefore, many adsorption peaks were observed. The peaks at 1590 and 3430 cm^−1^ corresponded to aromatic C=C and –OH, respectively. The adsorption band at 1120 cm^−1^ corresponded to the stretching vibration of the C–O bond. The signal at 592 cm^−1^ was the Fe–O adsorption peak, which indicated that the nZVI was oxidized on the BC [36].

These functional groups were believed to play a crucial role in the adsorption of ONZ and the support of nZVI. These functional groups immobilized nZVI and reduced the electrostatic attraction with nZVI [36]. A previous study indicated that nitroimidazoles can be adsorbed onto organic matter through interactions between the π electrons in the aromatic rings [37]. BC had abundant C=C and hydroxyl groups which acted as electron donors in the π–π aromatic interactions [33], while the nitro groups of ONZ acted as π-electron acceptors [38]. The π–π interactions enhanced the transfer of ONZ onto the surface of the BC, which increased the contact of ONZ and nZVI-BC.

### 3.2. Degradation of ONZ in Different Systems

**Figure 5:**
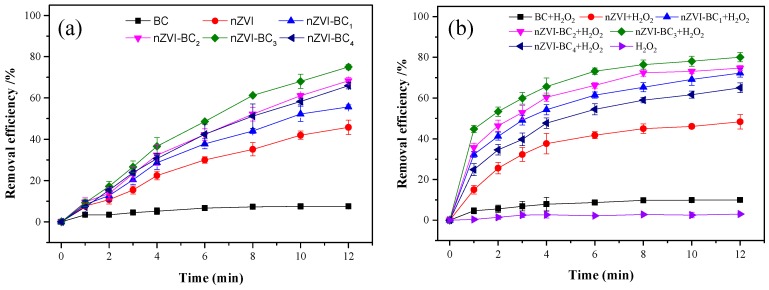
Comparison of ornidazole (ONZ) degradation for different systems: without H_2_O_2_ (**a**) and with H_2_O_2_ (**b**). Operating conditions: C_0_ = 100 mg/L, pH = 3.0, T = 25 °C, [H_2_O_2_]_0_ = 12 mM; BC = 0.2 g/L; nZVI = 0.1 g/L; nZVI-BC_1_ = 0.15 g/L (mass ratio of 2:1); nZVI-BC_2_ = 0.2 g/L (mass ratio of 1:1); nZVI-BC_3_ = 0.3 g/L (mass ratio of 1:2); nZVI-BC_4_ = 0.4 g/L (mass ratio of 1:3).

To examine the role of different materials, experiments were conducted in different systems, including BC, nZVI, and different nZVI-BC samples without H_2_O_2_. According to previous reports, ONZ is stable at a pH < 6 [8], and the result of the blank experiment was not presented. As shown in Figure 5a, the removal efficiency of ONZ was 45.8% in the nZVI system over 12 min, and the results were consistent with previous reports indicating that ONZ can be degraded by nZVI [39]. For the nZVI-BC system, the removal efficiency increased from 55.7% to 74.9% with an increase in the nZVI to BC mass ratio from 2:1 to 1:2. The results indicated that the BC played an important role in enhancing the activity of the nZVI [33]. As shown in Table 1, the BC had a larger specific surface area and total pore volume than the nZVI, and the attachment of the nZVI on the BC increased the dispersion and reduced the aggregation. Accordingly, the removal efficiency increased as the number of active sites increased. However, with a further increase in the mass ratio to 1:3, the removal efficiency decreased to 64.7%. This may be because the nZVI particles were enclosed by the excess BC [23,26], which hindered the contact of nZVI and ONZ.

In the experiment, approximately 7.5% of the ONZ in the BC was removed owing to the adsorption property of the BC. The variation of the pH in the degradation process was measured (Figure S1, Supplementary Materials). The pH increased from 3.0 to a final value of 3.5, indicating the corrosion of iron (Equation (9)). The pK_a_ value of the ONZ was 2.4 [40]. The ONZ was deprotonated and existed as an anionic species in the experiments. All the pH_PZC_ values of the catalysts were significantly higher than 3.5, and the surface was positively charged. Thus, the electrostatic interactions between the positively charged materials and anionic ONZ involved an attractive force. The adsorption behavior of the ONZ was mainly attributed to the π–π interactions and electrostatic attraction in our experiments, and the former was discussed in Section 3.1.

(9) $$\mathrm{Fe}+2H^{+}\to {\mathrm{Fe}}^{2+}+H_{2}.$$

Figure 5b shows the degradation of ONZ in the BC, nZVI, and different nZVI-BC composites in the presence of H_2_O_2._ The decomposition of ONZ was 2.2% in the H_2_O_2_ system, and approximately 9.9% of the ONZ in the BC/H_2_O_2_ system was eliminated, indicating that the BC was insufficient to induce H_2_O_2_ decomposition. For the nZVI/H_2_O_2_ system, H_2_O_2_ was activated with 48.3% ONZ removal. In contrast, the application of nZVI-BC enhanced the activation of H_2_O_2_, and >63% of the ONZ was removed in 12 min. The ONZ removal efficiencies were 72.3%, 76.6%, 75%, 80.1%, and 63.8% for nZVI/BC mass ratios of 2:1, 1:1, 1:2, and 1:3, respectively. Compared with nZVI, nZVI-BC had a larger surface area, which was beneficial for the distribution of nZVI and the adsorption of ONZ. Thus, the removal efficiency increased with the number of active sites. The results also indicated that nZVI-BC_3_ exhibited the highest removal efficiency; high BC content in nZVI-BC_4_ may cause the aggregation of BC sheets, which covered the reactive sites on the surface, reducing the removal efficiency [23,26]. Therefore, nZVI-BC_3_ was selected as the optimal composite in the subsequent experiments.

Although high ONZ removal efficiencies were obtained for both the nZVI-BC and nZVI-BC/H_2_O_2_ systems, more COD and TOC were removed in nZVI-BC/H_2_O_2_ than in nZVI-BC (Figure S2, Supplementary Materials). This may be because reduction was the removal mechanism in the nZVI-BC system [40], whereas, in the nZVI-BC/H_2_O_2_ system, a large number of carbon atoms in the ONZ molecules were converted into CO_2_ [7]. Similar results were obtained in previous studies [13]. Thus, nZVI-BC/H_2_O_2_ was selected as the optimal candidate system in this study.

### 3.3. Effects of Different Parameters on Degradation of ONZ

#### 3.3.1. Effect of Initial pH

**Figure 6:**
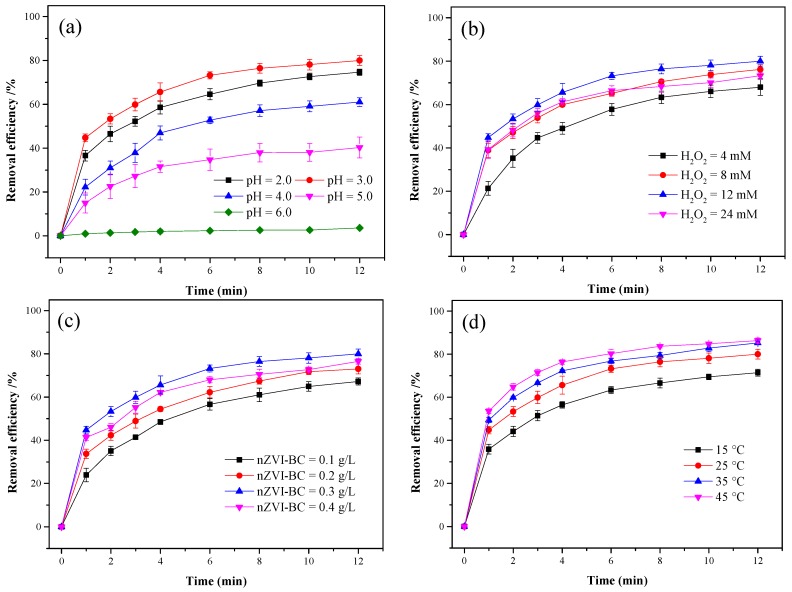
Effects of factors on the degradation of ONZ by nZVI-BC/H_2_O_2_: (**a**) initial pH; (**b**) H_2_O_2_ concentration; (**c**) nZVI-BC dose; (**d**) temperature. Except for the investigated parameter, other operation parameters were fixed as C_0_ = 100 mg/L, T = 25 °C, pH = 3.0, [H_2_O_2_]_0_ = 12 mM, and nZVI-BC_3_ = 0.3 g/L.

As shown in Figure 6a the degradation was significantly affected by the solution pH, and the optimal pH was 3.0. The removal efficiencies were 74.7% and 80.1% when the pH was 2.0 and 3.0, respectively. When the pH was <3.0, H^+^ atoms acted as •OH scavengers [41], and H_2_O_2_ was solvated to form a stable oxonium ion when a large number of H^+^ ions existed [42,43]. Consequently, the degradation efficiency at a pH of 2.0 was lower than that at a pH of 3.0. The removal efficiency decreased significantly as the initial pH increased. When the pH increased to 4.0 and 5.0, the ONZ removal efficiency decreased to 61.1% and 40.3%, respectively. At a pH of 6.0, no significant removal of ONZ was observed. Possible reasons for the foregoing findings are manifold. Firstly, the corrosion of Fe^0^ was faster and easier at lower pH values [32], which increased the concentration of Fe^2+^ and generated more •OH. Secondly, the oxidation potential of •OH was significantly influenced by the solution pH. At a higher pH, the oxidation ability of •OH was reduced [44]. Thirdly, the hydrolysis of iron ion at higher pH also had adverse effects on the formation of hydroxyl radicals due to the precipitation of FeOOH on the surface of nZVI [45,46]. Lastly, the iron–peroxo complex, which was the intermediate of Fenton reaction, becomes more stable with increasing pH and might yield less •OH for the degradation [47].

In conclusion, the pH significantly influenced the Fenton-like processes by controlling the catalytic activity of the iron species and the stability of H_2_O_2_ [41].

#### 3.3.2. Effect of Initial H_2_O_2_ Concentration

The effects of different initial H_2_O_2_ concentrations within the range of 4–24 mM were investigated. As shown in Figure 6b, H_2_O_2_ played a dual role in the degradation of ONZ. The ONZ removal efficiency increased from 68.1% to 80.1% when the H_2_O_2_ concentration increased from 4 to 12 mM, and it decreased from 80.1% to 73.3% when the H_2_O_2_ concentration increased from 12 to 24 mM. These results are consistent with previous reports [48]. At a low H_2_O_2_ concentration, the produced hydroxyl radicals were insufficient. With an increase in the initial H_2_O_2_ concentration, more H_2_O_2_ molecules could contact the nZVI-BC, leading to the generation of more •OH; thus, the removal efficiency increased. However, with excessive H_2_O_2_, excess H_2_O_2_ reacted with •OH and produced HO_2_• and O_2_• in accordance with Equations (10)–(12), which were more selective than •OH [49]. Therefore, the optimal H_2_O_2_ concentration for ONZ removal was 12 mM in the nZVI-BC/H_2_O_2_ system.

(10) $$•\mathrm{OH}+H_{2}O_{2}\to {\mathrm{OH}}_{2}•/O_{2}•+H_{2}O.$$

(11) $${\mathrm{HO}}_{2}•+•\mathrm{OH}\to H_{2}O+O_{2}.$$

(12) $$•\mathrm{OH}+•\mathrm{OH}\to H_{2}O_{2}.$$

#### 3.3.3. Effect of nZVI-BC dose

The effect of the nZVI-BC dose on the degradation of ONZ is shown in Figure 6c. The removal efficiency after 12 min increased from 67.2% to 80.1% when the dose increased from 0.1 to 0.3 g/L. However, with a further increase in the dose to 0.4 g/L, the removal efficiency decreased to 76.5%. The Fenton reaction was significantly affected by the ferrous irons provided by Fe^0^, which plays a major role in the formation of hydroxyl radicals [50,51]. The number of active sites increased with an increase in the nZVI-BC dose within the range of 0.1–0.3 g/L, leading to the release of more Fe^2+^ to react with H_2_O_2_ and the generation more •OH, which improved the removal efficiency. When the nZVI-BC dose further increased to 0.4 g/L, the ONZ removal efficiency may have decreased owing to the •OH scavenging effect of extra nZVI-BC through an undesirable reaction (Equation (13)) [14]. Consequently, the optimal nZVI-BC dose was 0.3 g/L in this study.

(13) $${\mathrm{Fe}}^{2+}+•\mathrm{OH}\to {\mathrm{Fe}}^{3+}+{\mathrm{OH}}^{-}.$$

#### 3.3.4. Effect of Temperature

The effect of the temperature was also investigated, and the results are shown in Figure 6d. The general trend was that increasing the temperature enhanced the removal of ONZ. The degradation efficiency increased from 71.4% to 86.3% when the temperature increased from 15 to 45 °C. A high temperature promoted the production of hydroxyl radicals [52] and enhanced the contact of ONZ molecules with nZVI-BC [53], leading to a higher degradation efficiency.

#### 3.3.5. Kinetics Study

According to previous studies [13,35], two kinetic models were used to analyze the organic compound degradation in the Fenton-like reaction (Figure S3, Supplementary Materials).

Pseudo-first-order reaction:

(14) $$\ln\left(\frac{C_{t}}{C_{0}}\right)=-k_{1}t.$$

Pseudo-second-order reaction:

(15) $$\frac{1}{C_{t}}-\frac{1}{C_{0}}=k_{2}t.$$

Here, k_1_ (min^−1^) and k_2_ (L∙mg^−1^∙min^−1^) represent the kinetic rate constants of the pseudo-first-order and pseudo-second-order reactions, respectively, and C_0_ and C_t_ represent the concentrations of ONZ (mg/L) at time t = 0 and at time t (min), respectively. The k values are presented in Table 2. The results indicated that the pseudo-second-order reaction model was more suitable for describing the degradation process (*R*^2^ > 0.95). The kinetic rate constants increased as the temperature increased. The activation energy of ONZ degradation was calculated using the Arrhenius equation.

(16) $$\mathrm{lnk}=-\frac{E_{a}}{\mathrm{RT}}+\mathrm{lnA},$$

where k (L∙mg^−1^∙min^−1^) represents the rate constant, E_a_ represents the Arrhenius activation energy (kJ/mol), A represents the Arrhenius factor, R represents the gas constant (8.314 J/(mol K)), and T represents the temperature. The Ea was calculated as 24.1 kJ/mol, indicating that the degradation of ONZ was a diffusion-controlled reaction [13]. It can be concluded that the degradation of ONZ in the nZVI-BC/H_2_O_2_ was easily achieved owing to the relatively low activation energy [54].

### 3.4. Stability and Reusability of nZVI-BC

**Figure 7:**
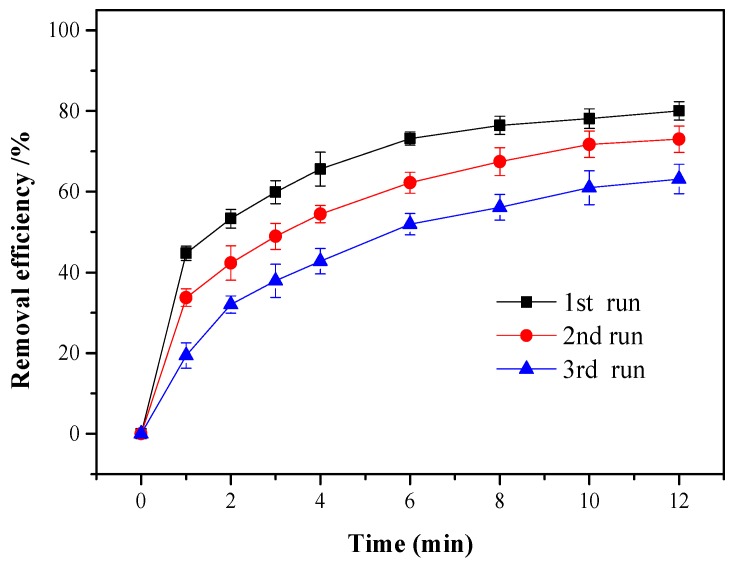
The recycling degradation of ONZ by the nZVI-BC/H_2_O_2_ system. Operating conditions: C_0_ = 100 mg/L, pH = 3.0, T = 25 °C, [H_2_O_2_]_0_ = 12 mM, nZVI-BC_3_ = 0.3 g/L.

The stability of nZVI-BC was investigated through an experiment involving consecutive cycles under the same conditions. After each cycle, the nZVI-BC was collected via vacuum filtration and washed several times for further use. As shown in Figure 7, the removal efficiency decreased from 80.1% to 63.2% after three recycling runs, owing to the release and consumption of Fe^0^ from nZVI-BC [23,24]. However, this was still a good value considering that a high concentration of the ONZ solution was treated in the experiments. In the future, efforts should be directed toward reducing the iron leaching and improving the recycling performance of nZVI-BC/H_2_O_2_ systems.

### 3.5. Possible Oxidation Degradation Mechanism

**Figure 8:**
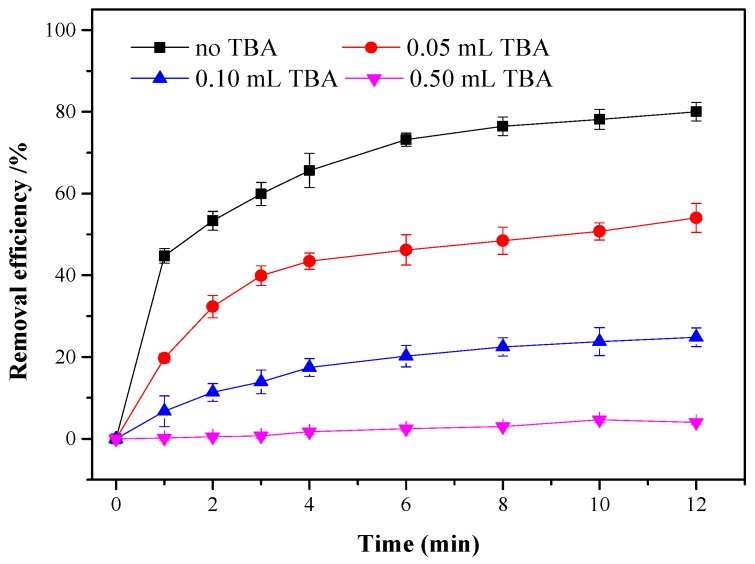
Degradation of ONZ with different *tert*-butyl alcohol (TBA) addition. Operating conditions: C_0_ = 100 mg/L, pH = 3.0, T = 25 °C, [H_2_O_2_]_0_ = 12 mM, nZVI-BC_3_ = 0.3 g/L.

The role of •OH in the degradation of ONZ was examined by conducting a control experiment in an H_2_O_2_ system with the presence of TBA. As shown in Figure 8, the removal efficiency decreased significantly with an increase in the TBA concentration. TBA is a good •OH scavenger and captures •OH during the Fenton reaction [55]. The removal of ONZ was almost completely inhibited with 0.5 mL of TBA. Therefore, the •OH played a decisive role in the oxidation of ONZ.

**Figure 9:**
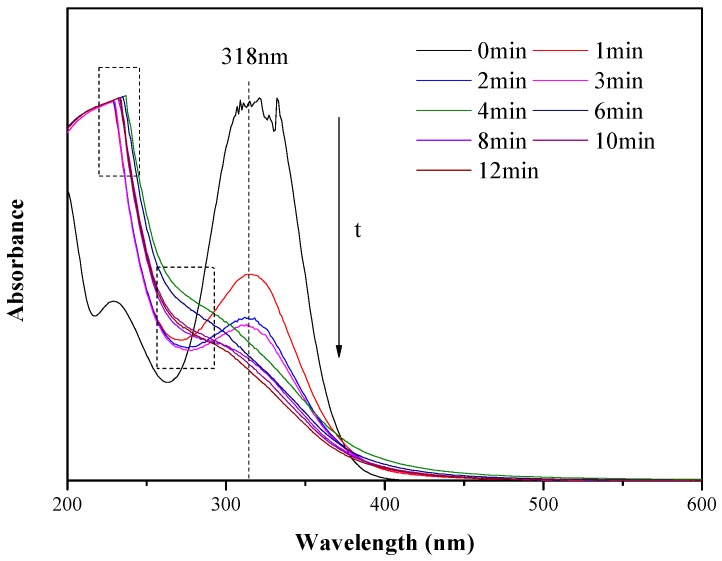
Ultraviolet–visible light (UV–Vis) spectrum of ONZ at various times. Operating conditions: C_0_ = 100 mg/L, pH = 3.0, T = 25 °C, [H_2_O_2_]_0_ = 12 mM, nZVI-BC_3_ = 0.3 g/L.

The UV–Vis spectrum of ONZ oxidation removal is presented in Figure 9. The characteristic absorption band of ONZ was approximately 318 nm and decreased with the increasing reaction time. A new adsorption band between 260 and 280 nm appeared and decreased, which may correspond to intermediates [56]. Furthermore, the peak between 220 and 240 nm increased significantly after the reaction started and remained relatively stable during the reaction, possibly indicating the formation of final products.

**Figure 10:**
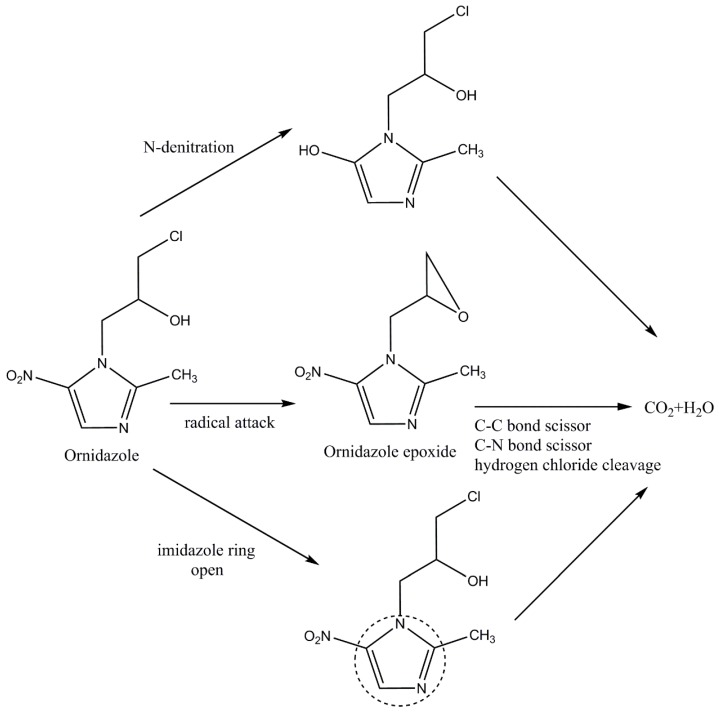
Possible pathways of ONZ degradation in the nZVI-BC/H_2_O_2_ system.

Nitrite, nitrate, and chloride ions were detected in the solution after the reaction (Figure S4, Supplementary Materials), indicating that these ions were formed in the oxidation process. The NO_2_^−^ ions are attributed to the N-denitration via radical attack [57], and they were released by radical substitution [31]. The NO_3_^−^ ions may have been related to the opening of the imidazole ring [57]. The Cl^−^ ions may have come from the formation of ornidazole epoxide via the attack of •OH on the ONZ [7] or the hydrogen chloride cleavage in the process of oxidation [8]. •OH is a strongly active non-selective agent, which was likely responsible for the scission of the C–N and C–C bonds [7]. According to these results, plausible pathways are proposed in Figure 10.

On the basis of these analyses, a possible mechanism of ONZ degradation in the nZVI-BC/H_2_O_2_ system is proposed. Firstly, the ONZ molecules were adsorbed onto the surface of nZVI-BC, and then Fe^2+^ was formed on the surface of iron nanoparticles via the corrosion of Fe^0^. Secondly, the H_2_O_2_ was activated by Fe^2+^, and highly reactive •OH was produced continuously. Finally, the •OH attacked the ONZ, and a part of the ONZ was mineralized into CO_2_ and H_2_O on the surface of nZVI-BC. Meanwhile, Fe^3+^ in the solution was gradually turned back and formed an oxidation layer on the Fe^0^ surface. In the process, the adsorption capacity and support ability of BC were fully exploited to improve the removal efficiency of ONZ.

Thus, the removal of ONZ can be described as follows:

(1) Adsorption of ONZ on nZVI-BC
  nZVI−BC+ONZ→nZVI−BC−ONZ.
(2) Generation of hydroxyl radicals
  $$\mathrm{Fe}+2H^{+}\to {\mathrm{Fe}}^{2+}+H_{2}.$$
  $${\mathrm{Fe}}^{2+}+H_{2}O_{2}\to {\mathrm{Fe}}^{3+}+•\mathrm{OH}+{\mathrm{OH}}^{-}.$$
  $$2{\mathrm{Fe}}^{3+}+\mathrm{Fe}\to 3{\mathrm{Fe}}^{2+}.$$
(3) Reaction of ONZ and hydroxyl radicals
  ONZ+•OH→intermediates.
  $$\mathrm{intermediates}+•\mathrm{OH}\to {\mathrm{CO}}_{2}+H_{2}O.$$

## 4. Conclusions

In this study, nZVI-BC was used as an activator for the Fenton-like oxidation of ONZ. Compared with nZVI and BC, the nZVI-BC had the highest removal efficiency for ONZ. The presence of BC in nZVI-BC not only enhanced the adsorption of ONZ but also provided more active sites. The results indicated that nZVI-BC_3_ (nZVI:BC = 1:2), which had the highest BET surface area, exhibited the best degradation performance among the samples tested. The effects of the initial pH, H_2_O_2_ concentration, nZVI-BC dose, and temperature on the degradation performance were examined in detail, and the degradation of ONZ followed a pseudo-second-order kinetics model (*R*^2^ > 0.95). The stability of nZVI-BC was also investigated, which exhibited a good performance even after three recycling runs. The mechanism of ONZ degradation involved the activation of H_2_O_2_ by nZVI-BC to generate •OH and the mineralization of ONZ into CO_2_ and H_2_O. Furthermore, the possible degradation pathways of ONZ degradation were proposed according to the variation of ions in the system. Overall, the study demonstrated that the nZVI/BC system has potential for removing ONZ from wastewater.

## Figures and Tables

**Table 1:** Characteristics of nanoscale zero-valent iron (nZVI), biochar (BC) and different nZVI-BC composites.

Name	Specific Surface Area (m^2^/g)	Pore Volume (cm^3^/g)
nZVI	12.56	0.0024
BC	227.45	0.1745
nZVI-BC_1_ (2:1)	62.03	0.1003
nZVI-BC_2_ (1:1)	73.12	0.1205
nZVI-BC_3_ (1:2)	89.93	0.1278
nZVI-BC_4_ (1:3)	86.39	0.1305

**Table 2:** The degradation rate constants and *R^2^* for different kinetic models. Operating conditions: C_0_ = 100 mg/L, pH = 3.0, T = 25 °C, [H_2_O_2_]_0_ = 12 mM, nZVI-BC_3_ = 0.3 g/L.

T (°C)	Pseudo-First-Order Model		Pseudo-Second-Order Model	
	k_1_ (min^−1^)	*R* ^2^	k_2_ (L∙mg^−1^∙min^−1^)	*R* ^2^
15	0.1033	0.8562	0.0021	0.9568
25	0.1336	0.8536	0.0035	0.9689
35	0.1482	0.8316	0.0045	0.9737
45	0.1605	0.8051	0.0055	0.9651

## Supplementary Materials

The following are available online at https://www.mdpi.com/1660-4601/17/4/1324/s1: Table S1. The Fe/C mass ratio and pHPZC of nZVI, BC and different nZVI-BC composites; Figure S1. The removal efficiency of ONZ and pH change during the degradation of ONZ. Operating conditions: C_0_ = 100 mg/L, pH = 3.0, T = 25 °C, [H_2_O_2_]_0_ = 12 mM, nZVI-BC_3_ = 0.3 g/L; Figure S2. The removal efficiency of COD and TOC in different systems. Operating conditions: C_0_ = 100 mg/L, pH = 3.0, T = 25 °C, (1) nZVI-BC system: nZVI-BC_3_ = 0.3 g/L; (2) nZVI-BC/H2O2 system: nZVI-BC_3_ = 0.3 g/L, [H_2_O_2_]_0_ = 12 mM; Figure S3. Plots of pseudo-first-order (a) and pseudo-second-order models (b). Operating conditions: C_0_ = 100 mg/L, pH = 3.0, [H_2_O_2_]_0_ = 12 mM, nZVI-BC_3_ = 0.3 g/L; Figure S4: The concentration of different ions. Operating conditions: C_0_ = 100 mg/L, pH = 3.0, T = 25 °C, [H_2_O_2_]_0_ = 12 mM, nZVI-BC_3_ = 0.3 g/L.
