# Supplementary material for: Fenton-Like Oxidation of Antibiotic Ornidazole Using Biochar-Supported Nanoscale Zero-Valent Iron as Heterogeneous Hydrogen Peroxide Activator
Source: Int J Environ Res Public Health. 2020 Feb 19;17(4):1324. doi: 10.3390/ijerph17041324 (PMC7068595; doi:10.3390/ijerph17041324)
Supplement: Supplementary file 1 [file ijerph-17-01324-s001.pdf]

Table S1. The Fe/C mass ratio and pH_{PZC} of nZVI, BC and different nZVI-BC composites.

Name	Fe (wt %)	C (wt %)	Fe/C	pH_{PZC}
nZVI	94.9	0.8	-	7.7
BC	0.4	80.2	-	5.1
nZVI-BC_1 (2:1)	50.8	27.7	1.8:1	6.8
nZVI-BC_2 (1:1)	40.2	43.8	1:1.1	6.5
nZVI-BC_3 (1:2)	30.1	56.2	1:1.9	6.1
nZVI-BC_4 (1:3)	21.6	62.9	1:2.9	5.9

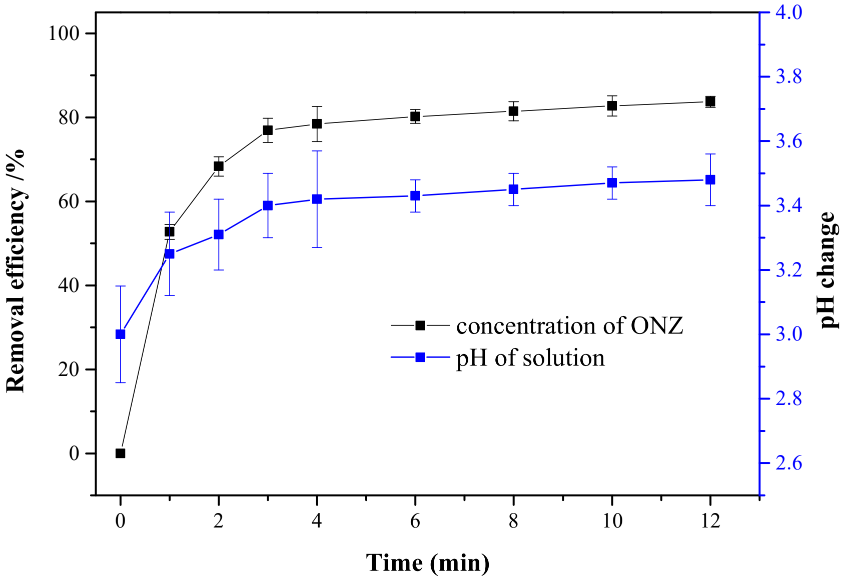

Figure S1. The removal efficiency of ONZ and pH change during the degradation of ONZ. Operating conditions: C_0 = 100 mg/L, pH = 3.0, T = 25 °C, $[H_2O_2]_0$ = 12 mM, nZVI-BC_3 = 0.3 g/L.

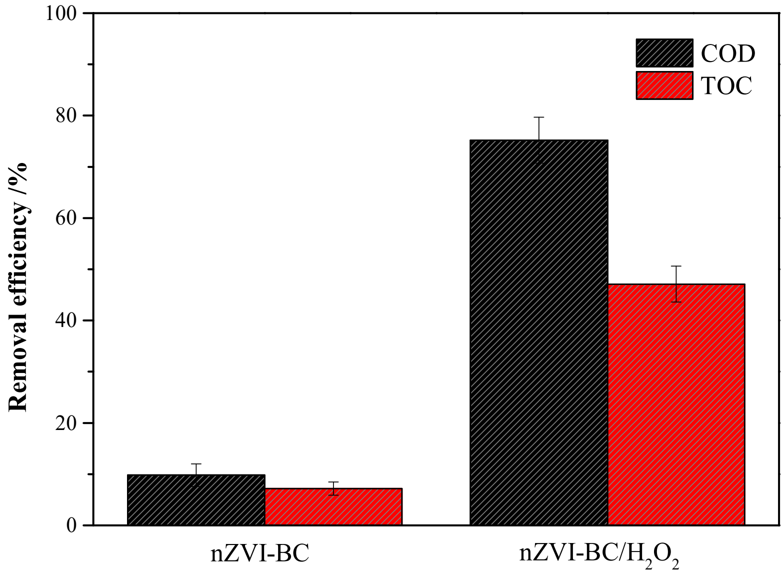

Figure S2. The removal efficiency of COD and TOC in different systems. Operating conditions: C_0 = 100 mg/L, pH = 3.0, T = 25 °C, (1) nZVI-BC system: nZVI-BC_3 = 0.3 g/L; (2) nZVI-BC/H_2O_2 system: nZVI-BC_3 = 0.3 g/L, $[H_2O_2]_0$ = 12 mM.

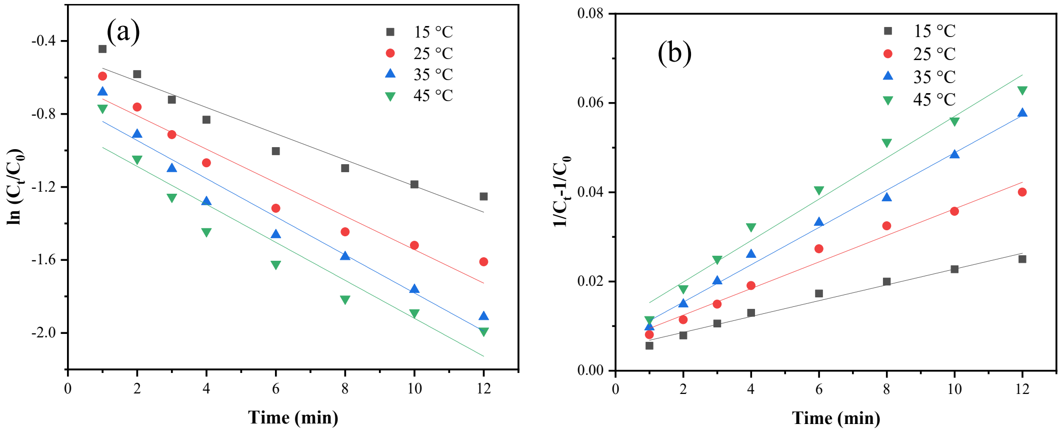

(**a**) (**b**)

Figure S3. Plots of pseudo-first-order (**a**) and pseudo-second-order models (**b**). Operating conditions: C_0 = 100 mg/L, pH = 3.0, $[H_2O_2]_0$ = 12 mM, nZVI-BC3 = 0.3 g/L.

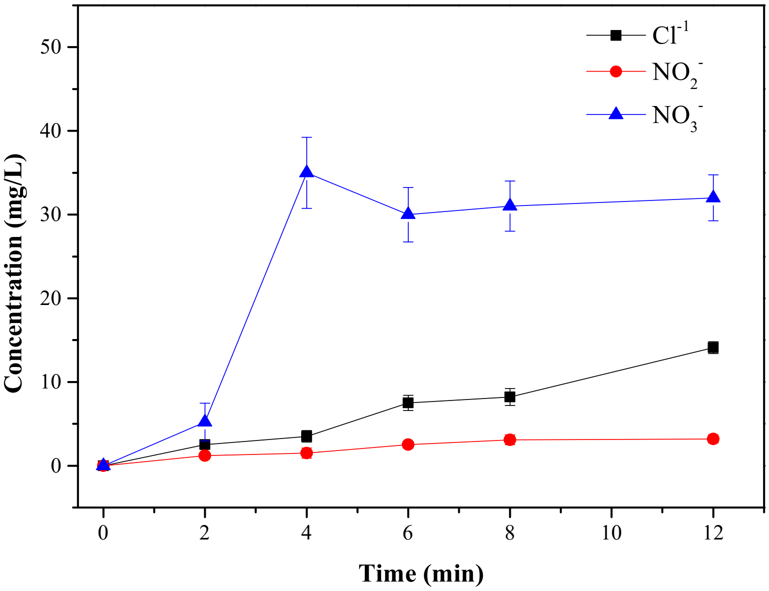

Figure S4. The concentration of different ions. Operating conditions: C_0 = 100 mg/L, pH = 3.0, T = 25 °C, $[H_2O_2]_0$ = 12 mM, nZVI-BC3 = 0.3 g/L.
